# Pituitary Macroadenoma Presenting as Acromegaly and Subacute Pituitary Apoplexy: Case Report and Literature Review

**DOI:** 10.7759/cureus.9612

**Published:** 2020-08-08

**Authors:** Artsiom Klimko, Cristina Capatina

**Affiliations:** 1 Division of Physiology and Neuroscience, Carol Davila University of Medicine and Pharmacy, Bucharest, ROU; 2 Department of Endocrinology, Carol Davila University of Medicine and Pharmacy, Bucharest, ROU; 3 Department of Pituitary and Neuroendocrine Pathology, C.I. Parhon National Institute of Endocrinology, Bucharest, ROU

**Keywords:** acromegaly, subacute, pituitary apoplexy, macroadenoma, subclinical

## Abstract

We report a case of a patient who presented to our endocrinology department for gradual onset with headache, fatigue, and weight loss over the course of one month. On physical examination, the patient showcased coarse facial features, acral enlargement, and other features suggestive of acromegaly. However, despite a clinical picture consistent with this diagnosis, serum growth hormone and insulin-like growth factor 1 were below reference range. Furthermore, secondary adrenal insufficiency, secondary hypothyroidism, and hypogonadotropic hypogonadism were discovered. Imaging revealed a pituitary macroadenoma and after a neurosurgical consult, the patient underwent transsphenoidal hypophysectomy and the suspected diagnosis of subacute pituitary adenoma apoplexy (SPAA) was confirmed via histology of resected tissue. Additionally, we review the literature for other case reports of patients with acromegaly or acromegalic features who underwent pituitary apoplexy to identify patient characteristics, presumed etiologies, and presence of biochemical cure of acromegaly following SPAA.

## Introduction

Acromegaly is a rare clinical syndrome driven by high serum values of growth hormone (GH) and insulin-like growth factor 1 (IGF-1). Functional pituitary adenomas are the most common cause of acromegaly, and the onset of the disease is characteristically gradual, leading to a substantial delay in diagnosis and treatment [1,2]. The purpose of this case report is to detail a rare complication of pituitary adenomas, namely subacute pituitary adenoma apoplexy (SPAA). In contrast to classic pituitary apoplexy, SPAA lacks the features of sudden onset severe headache, visual defects, or ophthalmoplegia, and can therefore present as a diagnostic challenge for clinicians [3,4]. Pituitary apoplexy can be associated with biochemical cure in secreting pituitary adenomas [5-7].

## Case presentation

A 41-year-old male patient was admitted to our endocrinology department for 8 kg weight loss over the past month, accompanied by loss of appetite, profound fatigue, decreased libido, and profuse sweating. He had a history of recurrent sinusitis and one month ago; there was a particularly protracted episode of left frontal and maxillary sinusitis requiring several courses of antibiotic treatment. He also complained of chronic headache that appeared periodically over the past five years.

Clinical examination revealed an asthenic patient with classic acromegalic features, namely mandibular prognathism, broad nose, thickened lips, prominent supraorbital ridges, and thickened skin folds on the scalp. These features were especially conspicuous when compared against photos over the last two decades. The patient also affirmed noticing enlargement of extremities, especially in the form of increasing shoe size, over approximately the last seven years. Surprisingly, paraclinical investigations (Table 1) revealed suppressed levels of GH and IGF-1 and rather than acromegaly, a diagnosis of pituitary insufficiency was made. Blood pressure was 120/60 mmHg without orthostatic hypotension, and heart rate was 56 beats per minute, without pathological changes on electrocardiography. The thyroid gland was also enlarged, and ultrasonography revealed multiple nodular cystic formations. Electrolytes and complete blood count values were within reference range.

**Table 1:** Laboratory results at admission. GH: growth hormone; TSH: thyroid-stimulating hormone.

Investigation completed	Reference range	Result of the investigation
Nadir growth hormone (post-oral glucose suppression test, ng/mL)	<1.000	<0.500
Insulin-like growth factor 1 (ng/mL)	55-235	167.5
Early morning cortisol (µg/dL)	5-25	<0.4
Fasting serum glucose (mg/dL)	70-99	67
TSH (µIU/mL)	0.5-4.5	0.059
Free T4 (ng/dL)	9-19	11.69
Prolactin (ng/mL)	2.5-17	0.92
Testosterone (ng/mL)	1.75-7.81	<0.1
Luteinizing hormone (mIU/mL)	2-12	<0.2
Follicle-stimulating hormone (mIU/mL)	1-12	0.7

**Figure 1:**
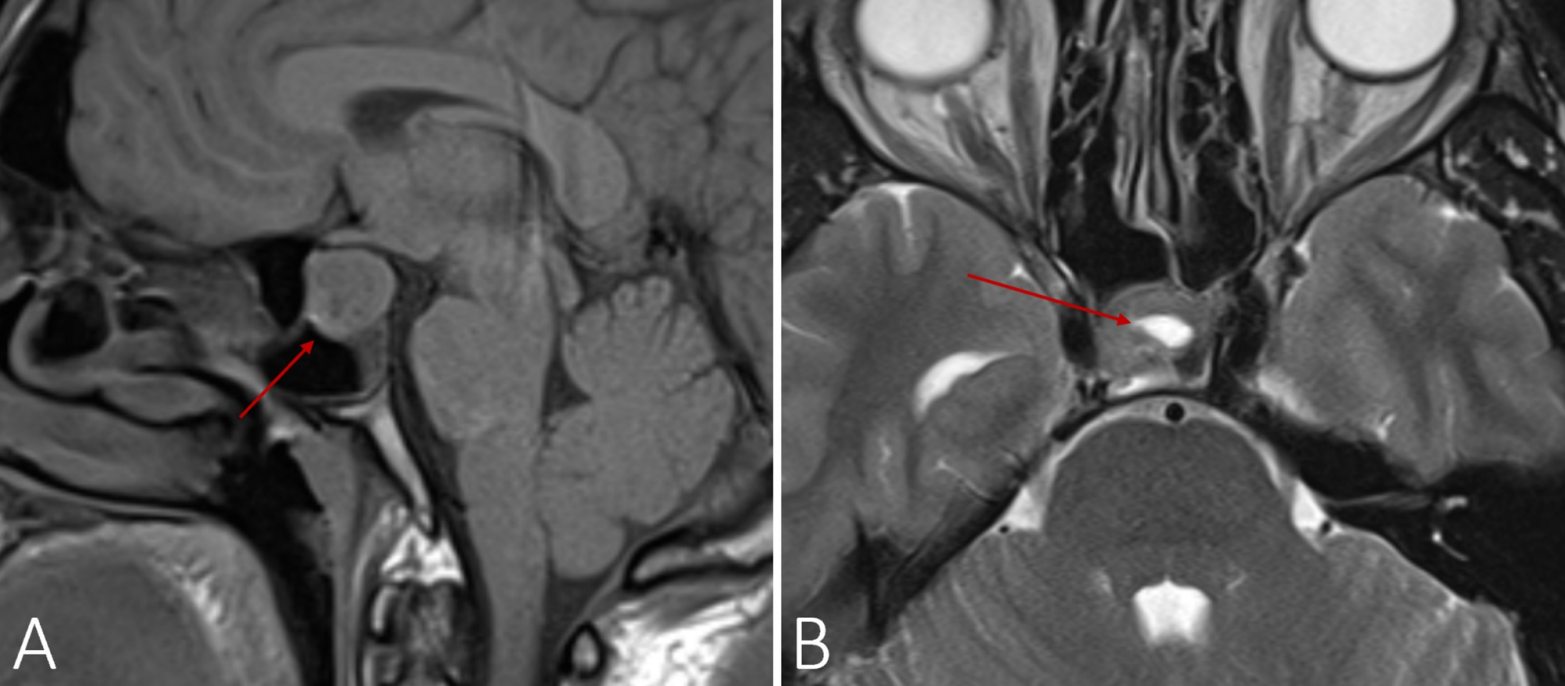
Cranial MRI: T1-weighted saggital plane (A) and T2-weighted axial plane (B) demonstrating a pituitary macroadenoma (214 mm x 168 mm x 147 mm); in the latter image, hyperintense filling (arrow) of the adenoma is also visible, suggesting pituitary apoplexy.

Cranial MRI revealed a pituitary macroadenoma (214 mm x 168 mm x 147 mm) with variable T1/T2 enhancement (Figure 1). The macroadenoma was not invading the cavernous sinus or compressing the optic chiasm and thus, the ophthalmologic exam (including the confrontational visual field testing) was normal. The patient was started on hormone replacement therapy with prednisone, levothyroxine, and testosterone undecanoate by intramuscular injection.

**Figure 2:**
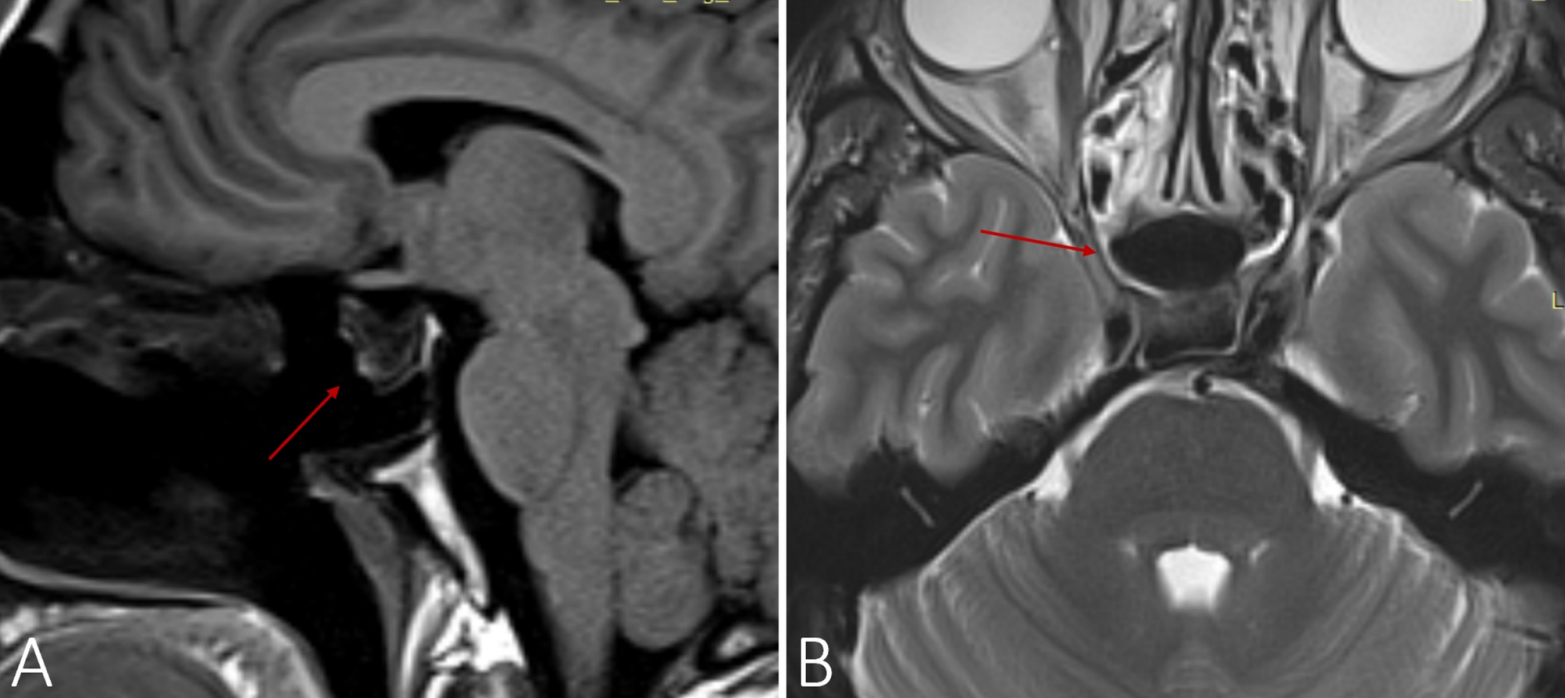
Cranial MRI: T1-weighted saggital plane (A) and T2-weighted axial plane (B) one year after transspnehoidal hypophysectomy, demonstrating an empty sella (arrow) and a confirming absence of new expansive lesions in the sellar region.

After starting replacement, glucose levels normalized, thus obviating the need to consider an insulinoma as part of multiple endocrine neoplasia type 1. Neurosurgical consult was requested and, one month later, elective transsphenoidal endoscopic resection was conducted for surgical debulking in view of the patient’s symptomatology, size of the adenoma, and presence of postero-superior hypothalamic compression. Histopathologic exam of the resected tissue revealed extensive coagulative necrosis and supported the diagnosis of pituitary apoplexy. The low prolactin levels also mirrored this, as low levels are suggestive of massive pituitary necrosis. The postoperative course was uneventful and at the latest follow-up visit 14 months after hypophysectomy, remission of most of the patient’s acromegalic features was noted. Control MRI did not show any new expansive lesions in the sellar region (Figure 2), as was confirmed by annual GH and IGF-1 assays.

## Discussion

In this case report, we presented a patient with subacute pituitary apoplexy leading to the biochemical cure of a previously undiagnosed GH-secreting pituitary adenoma and pituitary insufficiency. The pituitary necrosis was so extended that it completely destroyed the cells of the pituitary adenoma and made the precise diagnosis by biochemical tests or tumor immunohistochemistry impossible. In a recent review of population studies, Lavrentaki et al. found the annual incidence of acromegaly to range between 0.2 and 1.1 cases per 100,000 people, with median age of diagnosis ranging from 40.5 to 47 years, which is consistent with our patient [8]. GH-secreting pituitary adenomas are the most common cause of acromegaly and as recent evidence suggests, such tumors have wide clinicopathologic variation [2]. For example, sparsely granulated somatotroph tumors, which are found in 15%-35% of patients with acromegaly, are associated with subtle clinical features and relatively mild elevations in GH/IGF-1. This intertumoral heterogeneity explains why not all patients develop a rapidly progressive phenotype and provides insight into the substantial average diagnostic delay of eight years that is reported in many population studies [1,8].

Delay in diagnosis also likely explains why approximately 75% of somatotroph adenomas are detected as macroadenomas (adenoma > 10 mm) at the time of diagnosis [1,8]. This delay in diagnosis and expected tumor size were also findings consistent in our case. Long-term exposure to supraphysiologic levels of GH/IGF-1 leads to overgrowth of tissues, visceral organs, and systemic complications, like cardiovascular disease, which is the main cause of mortality [9]. Ezzat et al. conducted a prospective study on 500 patients and the clinical features with a prevalence over 50% of their cohort are presented in Table 2 [10]. Excessive acral growth, coarsening of facial features, and generalized soft tissue swelling were the most common features and all three were found in our patient, supporting the clinical diagnosis of acromegaly, despite the lack of confirmatory lab findings. We interpret the lack of biochemical confirmation in our case as the result of the extensive necrosis of the tumoral tissue. Current treatment options include transsphenoidal surgery, radiotherapy, or medical therapy with dopamine agonists, somatostatin analogs, or GH receptors antagonists. Although skeletal and articular changes are permanent, up to 70% of patients experience resolution of other systemic symptoms and metabolic complications [9]. 

**Table 2:** Common signs and symptoms of acromegaly and their presence/absence in our patient.

Prevalence	Signs and symptoms	Presence in our patient
98%	Acral growth	Yes
97%	Facial feature enlargement	Yes
90%	Soft tissue swelling	Yes
73%	Excessive perspiration	Yes
57%	Headache	Yes
53%	Peripheral neuropathy	No

The second component of this case is the appearance of SPAA. Classic pituitary apoplexy is defined as acute hemorrhage into the pituitary gland and as an entity, it is a life-threatening acute clinical syndrome, which, despite all the attention given to it, is exceedingly rare. In a retrospective analysis of 799 cases of pituitary adenomas, Bonicki et al. found 113 (14%) cases with histological evidence of infarction/necrosis and only a subset of 39 cases (34.5%) qualified for the diagnosis of pituitary apoplexy [4]. SPAA is more common and is defined by presence of hemorrhage and necrosis within the tumor in the absence of sudden onset severe headache, visual defects, or ophthalmoplegia [3,4]. In their retrospective analysis of 185 patients with SPAA, Zhang et al. noted the most common clinical presentation to include visual disturbances (76.7%), hyperprolactinemia (63.2%), and hypopituitarism (54.6%) [3]. However, in their cohort the majority of pathologies were constituted by prolactinomas (51.9%) and non-functional pituitary adenomas (37.0%), which is somewhat discordant with literature, where the most frequent preexisting tumor types are reported to be non-functioning pituitary adenomas, followed by prolactinomas and GH-secreting adenomas [5].

To further explore the association between acromegaly and pituitary apoplexy, a search of the PubMed and Scopus databases was done using the keywords “pituitary apoplexy,” “subacute apoplexy,” “subclinical apoplexy,” and “acromegaly” to identify relevant case reports published in the last 10 years. The keywords were identified in either title/abstract or as Medical Subject Heading (MeSH) terms. Of the 14 case reports found, 4 were excluded either due to being unrelated or written in a language other than English, resulting in 10 case reports which were included in our review and are presented in Table 3. The table structure was adapted from the article by Fraser et al. and including ours, 11 patients were reviewed [11].

**Table 3:** Cases published in the last 10 years describing co-occurrence of acromegaly and pituitary apoplexy. GH: growth hormone; IGF-1: insulin-like growth factor 1; IV: intravenous; GCS: Glasgow coma scale; CN: cranial nerve; DKA: diabetic ketoacidosis; FTM: female to male.

Author and date	Sex and age	Apoplexy type	Presenting signs and symptoms	Presumed trigger	Effect on GH/IGF-1	Effect on other pituitary hormones
Wildemberg et al., 2012 [12]	Male, 40	Acute	Severe headache and vomiting	Dengue hemorrhagic fever	No effect (elevated GH/IGF-1 at presentation)	Transient hypogonadotropic hypogonadism and hyperprolactinemia
Cinar et al., 2013 [13]	Male, 38	Acute	Severe headache, malaise, nausea, vomiting	Insulin therapy or MRI IV contrast	Decreased (GH 2.72 μg/L)	Panhypopituitarism (including diabetes insipidus)
Nganga et al., 2013 [6]	Female, 31	Subacute	One year of headache, irregular menses, and progressive decline in vision	Idiopathic	No effect (elevated GH/IGF-1 at presentation)	Hyperprolactinemia
Mir et al., 2013 [14]	Male, 63	Acute	Severe headache, vomiting, decreased consciousness (GCS 10), and right-sided CN III palsy	Idiopathic	Not available	Secondary adrenal insufficiency and central hypothyroidism
Jiang et al., 2013 [15]	Male, 49	Acute	Severe headache and DKA	Diabetes	No effect (elevated GH/IGF-1 at presentation)	No effect (all other hormones within reference range)
Villar-Taibo et al., 2014 [16]	Female, 51	Acute	Severe headache, nausea, vomiting, phonophobia, photophobia	Meningitis	Decreased (GH 0.6 ng/mL, IGF-1 72.5 ng/mL)	Panhypopituitarism (including diabetes insipidus)
Roerink et al., 2014 [17]	Male, 46 (FTM transformation)	Acute	Severe headache, nausea, vomiting, blurred vision	Idiopathic	No effect (elevated GH/IGF-1 at presentation)	Secondary adrenal insufficiency and secondary hypothyroidism
Roerink et al., 2015 [7]	Male, 41	Subacute	Episode of severe neck pain with fatigue and presyncope three months prior	Idiopathic	Decreased (GH 1.2 ng/mL, IGF-1 100.9 ng/mL)	Hypogonadotropic hypogonadism and partial secondary adrenal insufficiency
Kumar et al., 2016 [18]	Male, 18	Acute	Severe headache, vomiting, blurred vision	Idiopathic	No effect (elevated GH/IGF-1 at presentation)	Secondary adrenal insufficiency and secondary hypothyroidism
Patra et al. 2017 [19]	Male, 36	Acute	Severe headache	Polycythemia vera	No effect (elevated GH/IGF-1 at presentation)	Secondary adrenal insufficiency and secondary hypothyroidism
Current case	Male, 41	Subacute	Headache, fatigue, weight loss, decreased libido over past month	Idiopathic	Decreased (GH 0.05 ng/mL, IGF-1 167.5 ng/mL)	Secondary adrenal insufficiency, secondary hypothyroidism, and hypogonadotropic hypogonadism

The average age of the patients was 41.2 years (range 18-63), and of the eleven patients, nine were male and two were female. The clinical picture of the eight patients diagnosed with pituitary apoplexy was dominated by severe headache and nausea/vomiting. The proposed mechanism of pituitary apoplexy likely involves friability and increased fenestrations of neovasculature supplying the tumor, ischemia of the tumor due to mismatch between demand and supply, and compression of vessels supplying the tumor against the diaphragma sellae by the enlarging tumor [3,20]. This proposed mechanism, in combination with predisposing risk factors, is already well established but the precise trigger for apoplexy remains poorly understood and in the majority of the cases that were included in our review the precipitating factor was idiopathic. For the three patients with SPAA, the clinical picture is much less specific and especially if other confounding variables are present (such as testosterone therapy for gender transformation, as in the case report by Roerink et al.), the correct diagnosis can be more difficult to make [17].

For 10 patients, the values of GH and IGF-1 after apoplexy, but prior to undergoing surgery, were available and were not suppressed in six patients. We must stress that, in our patient, we did not find definitive biochemical proof of acromegaly, but this diagnosis is highly probably due to the presence of progressive clinical features which began to improve after the pituitary apoplexy event and surgical treatment. Analogously suppressed levels of GH and IGF-1 were found in two other case reports of SPAA [6,7]. Six patients still had increased levels of GH/IGF-1 and would be consistent with literature, where it is reported that the majority of GH-secreting pituitary adenomas remain functional after apoplexy [5]. All patients in our review were treated with transsphenoidal surgery, although patients with gradual evolution and a lack of severe, progressive neuro-ophthalmic signs (or other clinical signs which would be indications for neurosurgical decompression) can be managed conservatively.

## Conclusions

Patients with acromegaly or pathognomonic features may present with decreased levels of GH/IGF-1, and in these situations, a high index of suspicion must be maintained for subacute pituitary apoplexy by clinicians. Subacute pituitary apoplexy is more common than classic pituitary apoplexy, and a tepid emergence of non-specific symptoms, such as headache, fatigue, and weight loss, should tip clinicians off. The co-occurrence of pituitary insufficiency with SPAA is high and remission of acromegalic features in these patients is likely. However, for the patients in our review who presented with acute apoplexy, GH secretion was more likely to persist despite the apoplexy of the adenoma.
